# Extravasation Injuries: A Trivial Injury Often Overlooked with Disastrous Consequences

**DOI:** 10.29252/wjps.9.3.326

**Published:** 2020-09

**Authors:** Leon Alexander

**Affiliations:** Sheikh Khalifa Medical City, Division of Plastic Surgery, Department of Surgery, Abu Dhabi, UAE

**Keywords:** Extravasation, Iatrogenic injury, Necrosis, Infiltration, Saline

## Abstract

With the advent of parenteral, intravenous infusion for various purposes like chemotherapy, parenteral nutrition, radiocontrast intravenous injection for imaging studies, extravasation injuries are emerging as a serious problem with often disastrous complications, if not recognized early. Fortunately, if treated early, the affected extremities can be salvaged and hence the role of plastic surgeons cannot be over-emphasized, especially when it comes to the reconstruction of necrotic and ischemic wounds as a result of these injuries. Proper monitoring and immediate intervention will go a long way in minimizing the morbidity associated with these injuries. However, if there is a delay in recognition and treatment, it can lead to complications like skin necrosis, gangrene, extensive soft tissue defects and contractures. Treatment in these circumstances needs an individualized approach and entails wound debridement followed by skin grafts and flap cover. Documentation and prompt intervention can avoid medicolegal issues for the physician and the hospital.

## INTRODUCTION

Extravasation is the movement of fluid outside, and in the setting of an iatrogenic injury, it is usually a vesicant drug/solution from its conduit (vein) to the extracellular tissue environment with the resultant pressure effect leading to a local inflammatory reaction, compartment like syndrome, tissue necrosis, and skin breakdown. These iatrogenic injuries are becoming a regular occurrence, especially in the setting of intensive care unit (ICU), chemotherapy drug instillation, radiocontrast intravenous (IV) injection, and if not recognized early can have disastrous consequences like the loss of limb and function. The annual incidence of extravasation injury is only 0.1% to 0.7%, and it is 4.7-6.5% in the chemotherapy patient population and ranges from 11% to 58% in children.^1^^,^^2^ Gault described two techniques for the immediate treatment of these injuries namely the saline flush-out technique and liposuction in his series.^2^ We have described a modification of the saline flush-out technique, where stab incisions are used in grid-like pattern; thereby creating more channels for the extravasate to come out.

## CASE REPORT

A 6-month-old infant presented to us with extravasation injury of the dorsum of the right foot following IV infusion of 10% dextrose of which about 50 mL leaked out into the subcutaneous tissue of the right foot. This infant was a known case of Noonan’s syndrome with congenital heart disease and had undergone cardiac surgery. He was 15 days post-surgery, when this injury occurred; while he was being treated in the ICU for right lung collapse. Fortunately, the on-call plastic surgeons were called upon immediately, and we were able to intervene within 3-4 hours of the injury. 

On examination of the dorsum of the right foot, there was gross swelling of the foot and toes with blister formation, patchy areas of reddish-purple discoloration and pallor/whitening of the skin, and there was delayed capillary refill of toes, and distal dorsalis pedis pulse was not palpable due to edema. The distal sensation could not be assessed. As the child had multiple co-morbidities, a saline flush-out procedure was done on the bedside itself, after taking adequate sterile and aseptic precautions. 

After draping and prepping the foot, we used a modification of the saline flush-out technique, where a series of multiple stab incisions 2-3 mm in length were made and the depth of puncture was up to the subcutaneous level. These stab incisions were made with No: 11 scalpel blade in a grid-like pattern. Careful precautions were taken to avoid the areas of extensor tendons and neurovascular pedicle (dorsalis pedis artery and deep peroneal nerve), so as not to injure these critical structures. 

After completion of the stab incisions, a 10 mL syringe and 18 gauge cannula needle sheath were used for infiltration of saline into subcutaneous space, and the excess fluid was allowed to egress from the other incisions sites or expressed out manually by mild compression with a damp swab. This process of saline infiltration and flush-out was repeated until there was adequate relief of the intracompartment pressure as evidenced by a change in color of the foot, reduction in the size of gross swelling and improvement of capillary refill of toes. 

**Fig. 1:**
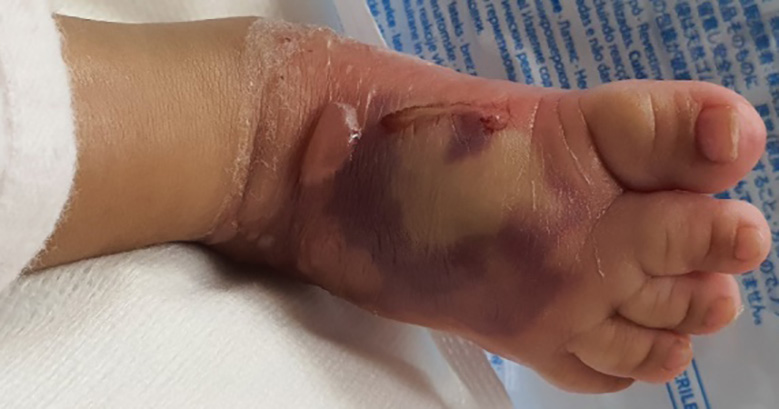
Extravasation injury of the dorsum of right foot following 10% dextrose IV infusion

**Fig. 2:**
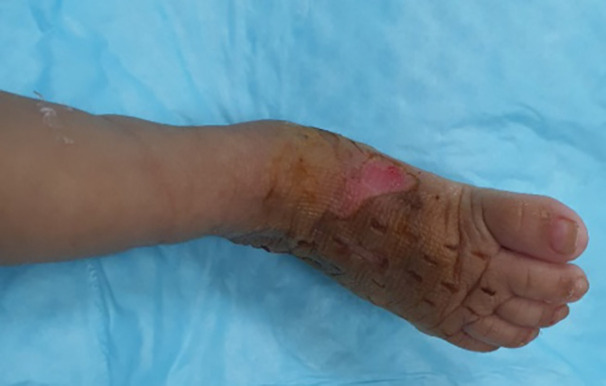
Appearance of the right foot after 1 week showing almost complete resolution with a small area of desquamation on the medial aspect which healed conservatively

During the process of saline flush out, hyaluronidase (1 mL diluted in 100 mL saline) was also used to facilitate the release of extravasated hypertonic dextrose solution. Approximately, 250 mL of saline was used to flush out the extravasated material. After this, a dressing was done with inadine, damp gauze, soft wool roll and crepe bandaging of the affected leg. The wound was reassessed the following day and showed good clinical improvement with reduction in swelling as well as good skin color, turgor and capillary refill. Figure 1 shows extravasation injury of the dorsum of right foot following 10% dextrose IV infusion and Figure 2 illustrates the appearance of the right foot after 1 week showing almost complete resolution with a small area of desquamation on the medial aspect which healed conservatively.

**Fig. 3:**
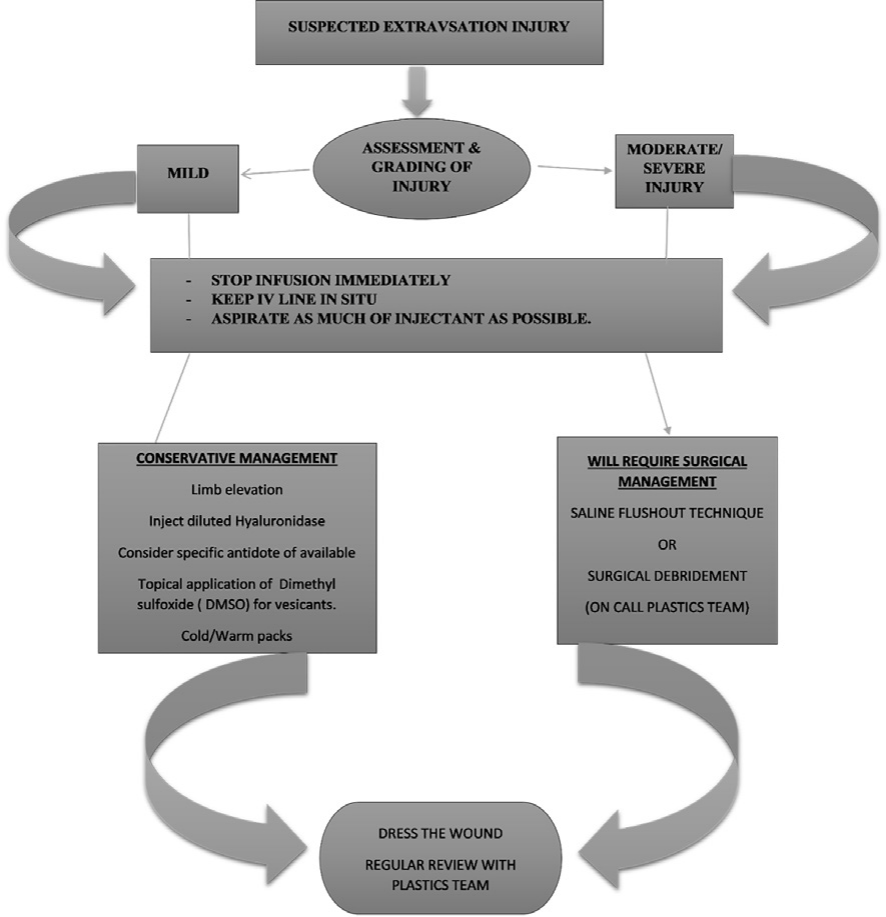
Algorithm proposed for the management of extravasation injuries based on the type (mild, moderate or severe) of injury

The dressing was repeated every day for a week, and after that, the patient was transferred to another facility and unfortunately was lost to follow up. Based on the clinical features, we can grade extravasation injuries as mild, moderate and severe, which will guide us in managing these injuries (Table 1). We have proposed an algorithm in the management of extravasation injuries based on the grade of injury too (Figure 3). All procedures followed were by following the ethical standards of the responsible committee on human experimentation (institutional and national) and with the Helsinki Declaration of 1975 (in its most recently amended version). The authors certify that they have obtained all appropriate patient consent forms. In the form, the patient has given the consent for the images and other clinical information to be reported in the journal. 

**Table 1:** Grading of extravasation injury

**Grading**		
**Mild**	**Moderate**	**Severe**
Minimal or no pain	Moderate pain and/or stinging	Severe pain and Singing
Mild singing	Skin blanching with/without discoloration	Skin blanching, pale with/without areas of reddish purple discoloration
Skin blanching/discoloration without edema	Skin blisters Edema	Skin blisters, breakdown/ulceration
Cool extremities	Cool extremities	Gross edema
No numbers	No signs of neurovascular compromise -no numbness, good capillary refill	Signs of neurovascular compromise and/or compartment syndrome – Numbness/absent sensation, poor/delayed capillary refill, pain of stretching of extremity

## DISCUSSION

A vesicant is a substance that can inflict severe and permanent tissue damage when extravasated. The extent of damage inflicted can be due to a compressive effect causing neurovascular compromise and direct cytotoxic effect, thereby causing symptoms of skin blanching, discoloration, erythema, swelling, pain, blister formation, vascular occlusion, inflammation, phlebitis, compartment syndrome and if allowed to progress unabated will lead to frank necrosis, gangrene, skin ulceration with soft tissue loss, compartment syndrome and the late sequel of delayed intervention being limb contractures (Volkmann’s ischemic contracture).^1^^-^^3^^,^^4^

Extravasation of acidic solution can cause skin necrosis and skin contractures, alkaline solutions like soda bicarb and hypertonic solutions (10% Dextrose) causes extensive subcutaneous damage, while extravasation of vasoconstrictive drugs like adrenaline can lead to ischemic necrosis.^1^ The soft tissue injury that occurs as a result of the extravasate is related to 4 factors including osmolarity, inherent cytotoxicity, infusion pressure, and vasoconstrictive properties of the injectant.^3^

The common type of extravasates implicated in these type of injuries included total parenteral nutrition (TPN contains amino acids, dextrose, lipids, electrolytes, vitamins, trace elements), phenytoin, dopamine, vinca alkaloids, anthracyclines, mechlorethamine (nitrogen mustard) and radiographic contrast materials. Prevention is the best cure in the treatment of these injuries, and the ideal way to effectively implement this strategy is the proper training and education of nursing and allied staff in proper technique and preventive strategies to minimize the occurrence of this complication.^3^

It is recommended that high-risk patients to be identified (neonates, elderly and comatose) and whenever peripheral IV infusions are started for these patients, there should be hourly monitoring of venous access sites for the signs of extravasation and if present appropriate help should be sought immediately.^3^ Treatment of these injuries should be emergent as “time is of the essence” and any delay in the identification and treatment can lead to unwanted complications. Hence, all suspected extravasation injuries are medical emergencies and should be approached in a systematic and logical manner with ultimate aim of minimizing the extent of damage.^5^^,^^6^

Several authors believe that urgent plastic surgery referrals are needed for all suspected extravasations, but we do not subscribe to this view as this can lead to a delay in a timely intervention for want of another specialist. We would like to emphasize that mild injuries can be managed by nursing staff supervised by the concerned doctors and only the moderate-severe injuries require expert opinion and appropriate action. Based on the grading system we have tabulated (Table 1), mild injuries can be managed conservatively, while moderate-severe extravasation injuries require surgical treatment which can be of 2 categories.

These categories include (i) saline irrigation and flush-out or liposuction; and (ii) surgical excision/debridement followed by delayed flap/skin graft cover. We have proposed an algorithm in the management of extravasation injuries based on the grade of injury. Some authors advocate aggressive surgical debridement of moderate-severe injuries in the emergent setting. However, such an approach is fraught with risks, as it is done before the actual line of demarcation sets in and can do more harm to the patient.^1^^,^^7^

However, it is always wiser to adopt a wait and watch approach, but only after an initial flush-out is done and if frank necrosis sets in, aggressive surgical debridement must be done to prevent its progression and further reconstructive surgery would be required depending on the depth and extent of involved structures. The conservative measures described for mild injuries include first and foremost to stop the infusion, retain the IV cannula and aspirate as much of the vesicant/injectant as possible, application of cold/warm packs (cold packs are contra-indicated for Vinca alkaloids), use of antidotes if available (dimethylsulfoxide, DMSO for anthracyclines and hyaluronidase), limb elevation and continuous monitoring.^1^^-^^3^^,^^7^

The local subcutaneous administration of hyaluronidase (1500 U diluted in saline) has been used to counter injury from several drugs including vinca alkaloids and anthracyclines.^1 ^Nevertheless, the treatment of extravasation injuries remains controversial, with no clear and universal guidelines to follow.^5^^,^^8^ Gault in his study reported that 88.5% of patients who were referred early were treated with a combination of liposuction and saline/hyaluronidase wash-out and suffered no complication; however, 52% of delayed referrals experienced extensive soft tissue damage and required reconstructive surgery (wound excision/debridement and flap/skin graft cover).^2^

Khan *et al.* in their series of 18 patients, 17 underwent early intervention with saline flush out technique with good outcome and only one patient required a delayed skin grafting for wound reconstruction.^9^ Amongst the methods described for surgical treatment of extravasation injuries the ‘saline flush out’ is the most commonly described with consistent and predictable outcomes if performed early.^1^^-^^3^^,^^5^^,^^9^^,^^10^ Gault first mentioned liposuction as a method for the treatment of extravasation injuries, where he did a combination of liposuction and saline flush-out technique in 6 patients and in one patient, liposuction was done without the use of tumescent.^2^

Several researchers described liposuction and saline lavage with good results and outcomes.^11^^-^^13^ There are many options available in the reconstruction of the extensive soft tissue defects as a result of these injuries ranging from simple secondary wound closure, split skin grafting (SSG) to more complex free microvascular tissue transfer. Ulcerations over the dorsum of the hand with exposed tendons would require a flap cover as a skin graft would stick to the tendon, thereby preventing proper gliding of tendons and finger movement.^4^^,^^14^^,^^15^

Options include pedicled abdominal flap, groin flap and free flaps like anterolateral thigh (ALT) flap, scapular flap, and lateral arm flap. For defects over the leg and dorsum of the foot with adequate soft tissue cover but only skin loss, an SSG was appropriate, but for extensive soft tissue loss, free flaps like ALT flap, groin flap, and scapular flap may be used.^2^^,^^4^^,^^9^^,^^14^^,^^15^ There have been reports of compartment syndrome occurring as a result of a missed or delayed presentation of extravasation injury, which then went onto ischemic necrosis, gangrene and later amputation of the extremity.^2^^,^^4^^,^^15^

## CONCLUSION

Extravasation injuries if intervened promptly can spare patients of unnecessary complications and reduce hospital stay as well as cost. There should be a low threshold for intervention by healthcare professionals in treating these injuries. With proper education and training of nursing and allied staff, the occurrence of these injuries can be reduced drastically. When cases are detected late or presenting with complications like skin necrosis leading to soft tissue defects with loss of function due to destruction of the underlying muscle, tendon, nerves and blood vessels, prompt referral to a plastic surgeon is imperative to salvage the extremity and prevent additional morbidities to such patients. 

## References

[1] Hahn JC, Shafritz AB (2012). Chemotherapy extravasation injuries. J Hand Surg Am.

[2] Gault DT (1993). Extravasation injuries. Br J Plast Surg.

[3] Hannon MG, Lee SK (2011). Extravasation injuries. J Hand Surg Am.

[4] D’Asero G, Tati E, Petrocelli M, Brinci L, Palla L, Cerulli P, Cervelli V (2010). Compartment syndrome of the hand with acute bullous eruption due to extravasation of computed tomography contrast material. Eur Rev Med Pharmacol Sci.

[5] Ghanem AM, Mansour A, Exton R, Powell J, Mashhadi S, Bulstrode N, Smith G (2015). Childhood extravasation injuries: improved outcome following the introduction of hospital-wide guidelines. J Plast Reconstr Aesthet Surg.

[6] Fallscheer P, Kammer E, Roeren T, Meuli-Simmen C (2007). Injury to the upper extremity caused by extravasation of contrast medium: a true emergency. Scand J Plast Reconstr Surg Hand Surg.

[7] Steiert A, Hille U, Burke W, Gohritz A, Zilz S, Herold C, Vogt PM (2011). Subcutaneous wash-out procedure (SWOP) for the treatment of chemotherapeutic extravasations. J Plast Reconstr Aesthet Surg.

[8] Goutos I, Cogswell LK, Giele H (2014). Extravasation injuries: a review. J Hand Surg Eur Vol.

[9] Khan MS, Holmes JD (2002). Reducing the morbidity from extravasation injuries. Ann Plast Surg.

[10] Thaha MA, McKinnell TH, Graham KE, Naasan AN (2007). Early intervention reduces morbidity in extravasation injuries from ‘lighter fuel’ injection. J Plast Reconstr Aesthet Surg.

[11] Steinmann G, Charpentier C, O’Neill TM, Bouaziz H, Mertes PM (2005). Liposuction and extravasation injuries in ICU. Br J Anaesth.

[12] Vanwijck R, Lengele B (1994). [Liposuction as a help for radiologists. Technical note]. Ann Chir Plast Esthet.

[13] Vandeweyer E, Heymans O, Deraemaecker R (2000). Extravasation injuries and emergency suction as treatment. Plast Reconstr Surg.

[14] Rudolph R, Larson DL (1987). Etiology and treatment of chemotherapeutic agent extravasation injuries: a review. J Clin Oncol.

[15] Alexander CM, Ramseyer M, Beatty JS (2016). Missed Extravasation Injury from Peripheral Infusion of Norepinephrine Resulting in Forearm Compartment Syndrome and Amputation. Am Surg.

